# A Social Media mHealth Solution to Address the Needs of Dengue Prevention and Management in Sri Lanka

**DOI:** 10.2196/jmir.4657

**Published:** 2016-07-01

**Authors:** May O Lwin, Santosh Vijaykumar, Vajira Sampath Rathnayake, Gentatsu Lim, Chitra Panchapakesan, Schubert Foo, Ruwan Wijayamuni, Prasad Wimalaratne, Owen Noel Newton Fernando

**Affiliations:** ^1^ Centre of Social Media Innovations for Communities Nanyang Technological University Singapore Singapore; ^2^ Nanyang Technological University School of Computer Science and Engineering Singapore Singapore; ^3^ Colombo Municipal Council Colombo Sri Lanka; ^4^ University of Colombo School of Computing Colombo Sri Lanka

**Keywords:** dengue, public health inspector, mhealth, social media, surveillance, needs assessment, prevention and management

## Abstract

**Background:**

Sri Lanka has witnessed a series of dengue epidemics over the past five years, with the western province, home to the political capital of Colombo, bearing more than half of the dengue burden. Existing dengue monitoring prevention programs are exhausted as public health inspectors (PHIs) cope with increasing workloads and paper-based modes of surveillance and education, characterizing a reactive system unable to cope with the enormity of the problem. On the other hand, the unprecedented proliferation and affordability of mobile phones since 2009 and a supportive political climate have thus far remained unexploited for the use of mobile-based interventions for dengue management.

**Objective:**

To conduct a needs assessment of PHIs in Colombo with respect to their dengue-related tasks and develop a new mobile-based system to address these needs while strengthening existing systems.

**Methods:**

One-on-one in-depth interviews were conducted with 29 PHIs to a) gain a nuanced, in-depth understanding of the current state of surveillance practices, b) understand the logistical, technological and social challenges they confront, and c) identify opportunities for mobile-based interventions. Quantitative analysis included simple descriptive statistics while qualitative analysis comprised textual analysis of 209 pages of transcripts (or nearly 600 minutes of conversations) using grounded theory approaches.

**Results:**

Current paper-based data collection practices for dengue surveillance involved a circuitous, time consuming process that could take between 7-10 days to officially report and record a single case. PHIs confronted challenges in terms of unreliable, standalone GIS devices, delays in registering mosquito breeding sites and lack of engagement from communities while delivering dengue education. These findings, in concert with a high motivation to use mobile-based systems, informed the development of Mo-Buzz, a mobile-based system that integrates three components – digitized surveillance, dynamic disease mapping and digitized dengue education – on a common platform. The system was developed through an iterative, evolutionary, collaborative process, consistent with the Spiral model of software development and is currently being used by all 55 PHIs in the CMC system.

**Conclusions:**

Given the entrenched nature of existing paper-based systems in PHIs’ work habits, we expect a gradual adoption curve for Mo-Buzz in the future. Equally, we expect variable adoption of the system with respect to its specific components, and specific PHI sub-groups (younger versus older). The Mo-Buzz intervention is a response to multiple calls by the global mHealth community for collaborations in the area of mobile interventions for global health. Our experience revealed that the benefits of this paradigm lies in alleviating country-specific public health challenges through a commonly shared understanding of cultural mores, and sharing of knowledge and technologies. We call upon future researchers to further dissect the applicability of the Spiral Model of software development to mHealth interventions and contribute to the mHealth evidence debate from theoretical and applied perspectives.

## Introduction

Dengue, the vector borne disease that threatens the lives of millions of people in tropical countries, has severely affected Sri Lanka in the past 2 decades. In 2014, the country reported nearly 40,000 dengue cases, a level of burden that has been consistent over the past few years [1]. Curiously, more than 55% of such cases were found to originate from the western province of Colombo, where the country’s political capital is located.

Given that the severity of dengue outbreaks has failed to abate, Sri Lanka, and more specifically, the capital city of Colombo, grapples with an exhausted dengue outbreak management system. Dimensions of the systemic fatigue are sporadically highlighted in the mainstream media, such as the case of the public health inspectors (PHIs)—the last mile in Sri Lanka’s public health delivery system—who are overtly burdened to an extent of one PHI covering a population of nearly 50,000 citizens [2]. Beyond dengue surveillance, the PHI’s daily duties include contributing to the control of other communicable and noncommunicable diseases, reporting on housing and sanitation issues, water supply and waste control, adolescent and reproductive health, and health education and promotion among others. It is reasonable to assume that this wide-ranging job description places undue demands on, and adversely affects, the efficacy of the dengue management system in Colombo. To our knowledge, no research studies have examined occupational challenges faced by PHIs in Colombo, as there have been on rural health workers in India, Vietnam, and sub-Saharan Africa [3-8]. By similar accounts, there is limited evidence critically examining their specific role in the vector management system in a way that gives us a glimpse into opportunities for potential interventions to enhance the effectiveness of prevention programs.

### Role of Mobile Technology in Dengue Prevention

Sri Lanka has witnessed an unprecedented growth in the penetration of mobile services after 2009 when the civil war ended. Currently, Sri Lanka boasts one of the most affordable rates of mobile services across the world, with penetration rates higher than most developing countries [9]. These developments are reflected in national corporate and governmental policies that have together initiated a series of Mobile for Development (or M4D) programs with health and education serving as priority areas [10]. However, dengue programs have yet to benefit from this technological trend, even as vast swathes of the Sri Lankan population become increasingly susceptible to this vector-borne disease.

In other developing countries in tropical regions, technological interventions for bolstering dengue surveillance have mainly focused on the use of geographical information systems (GISs) and other surveillance systems to facilitate early notification or warnings of potential outbreaks. For instance, Chang and colleagues [11] used Google Earth and ArcGIS 9 to create a surveillance system in Nicaragua that can allow public health workers to identify high indices of mosquito infestation in relation to larval development sites like garbage piles and stagnant water pools. In Brazil, researchers developed the SMCP-Aedes, an entomological surveillance system focused on collecting, storing, analyzing, and disseminating mosquito-related information on the Web [12]. In Thailand, Ditsuwan and colleagues [13] used a combination of a national surveillance system database and GIS to evaluate the burden of dengue and chikungunya fever. Dengue-GIS has also been used for monitoring and evaluating national-level epidemiological, entomological, and control interventions in Mexico and has been found to be useful for decision making at different levels of the dengue control system [14]. Although these initiatives have attempted to use GIS for different aspects of dengue prevention and control programs in their respective countries, we recognize 3 main limitations in extant work. First, we notice a paucity of technological interventions that reach beyond the offices of health policymakers and authorities to influence the actual workflow of health workers at points where they interact with the public. Second, it is evident that most technological interventions are focused mainly on surveillance but rarely facilitate efficient health education or community engagement, two of the bedrocks of any powerful dengue prevention program [15,16]. Third, the cost-effective nature and multifunctional capabilities of mobile phones have been used for a range of public health concerns in developing countries [17], but not as much for dengue.

On the basis of the previously mentioned review of literature, our exploratory study was guided by the following aims: (1) to gain a comprehensive understanding of the epidemiological process of identifying, reporting, and recording dengue cases in Colombo, (2) to understand the PHI’s logistical, technological, and social risks and challenges in the processes identified in (1), (3) to identify opportunities for technological intervention based on existing beliefs about and familiarity with mobile technologies among PHIs, and (4) to develop a technological intervention that can address the most critical gaps in their existing workflow to enhance the overall efficiency of the dengue management system in Colombo.

Our paper is presented in 3 main sections. First, we present results from a mixed-methods technological needs assessment of PHIs in Colombo. Second, we present a detailed description of a social media–based system, called Mo-Buzz, which we developed to address the most critical bottlenecks in the current *paper-based* dengue information system. The final section culminates with a discussion of study findings, implications of such a system for the larger public health infrastructure in Colombo, and potential future research.

## Methods

To assess the dengue-related informational and technological needs of the PHIs in Colombo, we conducted a series of in-depth interviews with them that would allow us to gain a nuanced, multifaceted perspective in the issues of utmost concern to them. Each interview was preceded by a simple quantitative survey. We next present details of each of the survey and the in-depth interviews.

### Survey

The survey was designed to obtain a demographic profile of the PHIs; examine their technological habits, usage, and preferences in the dengue context; and generally assess their readiness to move forward and adopt and use the technology. The questionnaire comprised sections on measurement of demographic variables such as age, ethnicity, education, income, and years of experience as a PHI. We then captured technology use through a simple yes or no question asking whether they have previously used the Internet, simple mobile phone, a smartphone, mobile apps, and a tablet. Descriptive analyses were conducted using univariate statistical techniques on SPSS, v. 21 (IBM Corp. Armonk, NY).

### In-Depth Interviews

The in-depth interviews complemented the survey and were aimed at getting a deeper, more detailed, and nuanced perspective on the PHIs role in the public health system, their specific functions related to dengue prevention and management, and their beliefs on the potential for technological interventions in their dengue-related duties. An interview guide facilitated the flow of the conversation across the following themes: (1) roles and responsibilities of the PHI, (2) perspectives on the dengue burden in Colombo, (3) data collection and information flow pertaining to dengue monitoring and surveillance and challenges in this process, (4) health educational activities related to dengue, (5) technology use and preferences, (6) ideas for technological intervention, and (7) perspectives on client (the general public or community members whom the PHIs interface with on a daily basis) interaction, client trust, and client satisfaction. All interviews were conducted in Sinhalese or Tamil (the major local languages). All interviews were digitally recorded and later translated into English by an experienced translator.

Data were collected through a series of detailed one-on-one interviews with the PHIs at the office of the Colombo Municipal Council (CMC). All interviews were conducted with the permission and approval of the Chief Medical Officer of the CMC who also assured access to the PHIs. The Chief Medical Officer briefed the cadre of PHIs about the interviews (before the commencement of the study) and set up a schedule where every PHI would be scheduled to attend a 30- to 45-minute time slot at the interview venue based on their schedule. At the start of each session, the research staff described the aims and purpose of the study to the PHI and obtained their signatures on an informed consent form after explaining the terms of the study including data confidentiality. Each study comprised a short 10-minute quantitative component followed by an in-depth interview (qualitative) that lasted between 30 and 60 minutes.

Qualitative data analysis was conducted through grounded theory approach in 2 ways. First, analysis proceeded as data were collected, thereby allowing early findings to influence later inquiries. For instance, when we observed that the first few participants alluded to contextual constraints associated with dengue-related data collection on the ground (a subtopic not originally part of our interview guide), we gradually introduced this theme into subsequent interviews to explore this area even more. Second, emergent findings and a review of the transcripts were used to design a preliminary coding guide that was further refined as coding and analysis proceeded. Two researchers coded each transcript independently, and then arrived at a final code after discussing their codes with an adjudicator. The final codes were then processed through NVivo, a software that helped to summarize qualitative data using visual and tabular formats, analyzing frequency and prominence of topics discussed by the PHIs. Eventually, the research team coded 585 minutes of interviews spread over 5 days and coded 209 pages of interview transcripts.

## Results

### Survey

As seen in Table 1, the 29 PHIs whom we interviewed were nearly equally distributed between the 21-30 years and 31-40 years age groups with all but one belonging to Sinhalese ethnicity. Nearly 86% (25/29) of them had attained a diploma. Nearly 45% (13/29) of the PHIs had served their role for less than a year, nearly 21% (6/29) between 1 and 5 years and the remaining 35% (10/29) were regarded as seniors having served more than 5 years.

**Table 1:** Demographic breakdown of the PHIs.

Category		Frequency (N)	Percentage (%)
Gender			
	Male	29	100.0
Age			
	21-30	16	55
	31-40	13	45
Ethnicity			
	Sinhalese	28	96
	Indian Tamil	1	4
Highest educational level			
	Secondary and below	3	10
	Certificate or diploma	25	86
	University and above	1	4
Duration of service			
	Less than 1 year	13	45
	1-5 years	6	21
	More than 5 years	10	35
History of digital technology use			
	Internet	25	86
	Simple mobile phones	26	90
	Smartphones	18	62
	Mobile apps	10	35
	Tablets	8	28

Our descriptive analysis revealed a healthy history of technology use with nearly 86% (24 of 28) of the PHIs having used the Internet and 90% (25 of 28) having used simple mobile phones. Of these, only 62% (17 of 28) of them had used smartphones previously, and a mere 28% (8 of 28) had prior experience with tablets. Finally, nearly 35% (10 of 28) of the PHIs were familiar with mobile apps.

An analysis of the constructs (Table 2) revealed interesting insights. Although perceived ease of using [18] mobile apps and tablets was relatively low with means of 3.63 and 3.47, respectively, their perceived usefulness [18] for dengue collection was among the highest, with means of 3.52 and 3.86, respectively. In addition, the PHIs reported that better technology would strengthen their ability to track (mean [M]=4.52, standard deviation [SD]=.63) and report (M=4.69, SD=.71) dengue cases more efficiently and make it easier for them to identify new mosquito breeding sites (M=4.28, SD=.92).

**Table 2:** Technology-related attitudes.

Constructs		M	SD
Perceived ease of use			
	Internet	4.28	.88
	Simple mobile phones	4.77	.62
	Smartphones	4.34	.80
	Mobile apps	3.63	1.10
	Tablets	3.47	1.10
Perceived usefulness			
	Paper and pen	3.41	1.46
	Simple mobile phones	2.79	1.43
	Smartphones	3.54	1.03
	Mobile apps	3.52	1.15
	Tablets	3.86	1.23
Perceived utility for dengue tasks			
	I can easily track new dengue cases in Colombo	3.41	1.02
	I can easily report new dengue cases in Colombo	3.61	1.01
	I can easily identify new mosquito breeding sites in Colombo	3.24	1.02

### In-Depth Interviews

In this section, we first present a description of the existing paper-based surveillance process as reported by the PHIs and then examine specific issues of interest to our technological development process.

#### Understanding How Dengue Cases Are Identified, Reported, and Stored in Colombo

**Figure 1:**
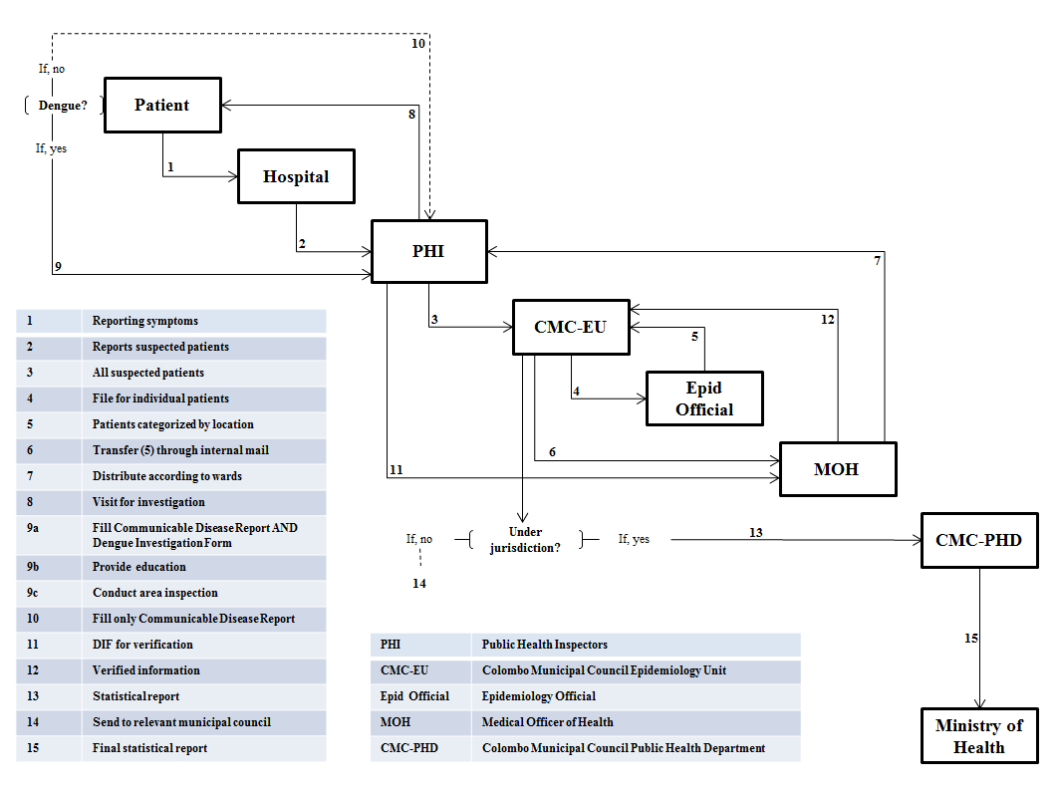
Existing flow of dengue information in Colombo, Sri Lanka.

We obtained a comprehensive understanding of the flow of dengue-related information between different agencies involved in the dengue surveillance programs in Colombo. As shown in Figure 1, the existing dengue information architecture reflects a circuitous and time-consuming process. This process commences with a patient who experiences symptoms visiting the hospital who in turn hand over a paper-based record of suspected dengue cases to the PHI who is assigned to that particular hospital. All PHIs who receive this information pass it along to the CMC Epidemiological Unit (CMC-EU), where an official is assigned to create a separate file for individual patients. The official categorizes all these files according to the Medical Officer of Health (MOH) jurisdiction under which they are covered and dispatches this information to each of the MOH offices through the CMC-EU. The MOHs then distribute the files to their PHIs for follow-up through patient visits. Each PHI visits the patient to confirm his or her diagnosis for dengue, on which a decision is taken to fill either a Communicable Disease Form (CDF) and a Dengue Investigation Form (DIF) or only the former, depending on whether the patient is tested positive or negative. In addition, in case of a positive diagnosis, the PHI is required to conduct a house and area inspection to identify possible mosquito breeding sites and educate the patient and his family on protecting themselves from dengue. After obtaining the entire set of CDFs and DIFs from the PHIs under their jurisdiction, the MOHs officially approve the forms before dispatching them to the CMC-EU. The CMC-EU manually collates the information from all the DIFs to create a record, map dengue cases on a manual map, and ensure that all the cases are within the CMC jurisdiction. At the end of this process, a formal report is sent to the CMC Public Health Department who officially sign on it before dispatching it to the Ministry of Health. The whole process could take anywhere from 7 to 10 days.

We now examine specific issues of interest. Table 3 tabulates the distributions of each topic mentioned by PHIs and their prevalence in the overall discussion.

**Table 3:** Distribution of topics.

Topic	Percentage of PHIs who discussed a specific topic (%)	Percentage of reference in relation to overall references (%)	Percentage of reference in relation to overall conversation size (%)
Barriers impeding PHI’s work	100.0	13.6	16.7
Epidemiology about dengue	100.0	9.4	13.6
Process of PHI’s work	100.0	19.4	21.1
Prevention of dengue	96.4	5.4	8.9
Knowledge about dengue (PHIs)	92.9	5.1	7.0
Education materials about dengue	89.3	8.0	9.3
Attitude of public towards PHIs	86.0	6.4	6.0
Equipment used for dengue tasks	85.7	5.6	8.0
Profile of PHI	85.7	2.5	7.7
Suggestion for mobile app	85.7	6.3	7.6
Responsibility of PHIs	78.6	4.4	4.5
Facilitators to PHI’s work	71.4	2.6	3.8
Client interaction with PHIs	68.0	3.0	6.0
Burden of dengue	67.9	2.0	3.0
Knowledge about dengue (public)	67.9	1.9	2.9
Trust among clients on PHIs	50.0	1.4	1.4
Story from PHI’s work life	46.4	1.0	3.5
Diseases related to mosquitoes	39.3	0.9	2.8
Technology use of PHIs	25.0	0.4	0.5
Demographic factors	14.3	0.4	0.4
Client satisfaction	10.7	0.3	1.0

The interviews revealed that the most complex and challenging sub-processes from the process (presented in steps 8 and 9 of Figure 1) involved PHIs field visits to the residences of potential dengue patients and the follow-up actions. The other steps mostly involved manual transfer of documents from one set of actors to the other within the system, but steps 8 and 9 involved multiple logistical, technological, and social elements that infused tension in the system. As such, these steps are the bedrock of the dengue data surveillance in Colombo and thus required most attention. Here, we outline practical challenges faced by PHIs while executing their dengue data collection tasks.

#### Logistical Challenges

The foremost uncertainty for the PHIs arose when they would attempt to visit the residences of the prospective dengue patients, as assigned by their respective MOHs. The PHIs noted multiple instances when the patients would either be away or would have relocated, rendering their visit futile. In some cases where it would be challenging for the PHI to locate the client’s exact address, the PHI would be unable to contact them and inquire as the patient would have refused to share their mobile number on account of privacy issues. If the clients were available, the PHIs would have to complete the requisite procedures, fill up the lengthy forms, and then commute to the respective office to submit them. A combination of these factors contributed to delays in data collection ultimately reducing systemic efficiency. The laboriousness of the process was highlighted by PHI 23 when he noted:

I would say finding the patients is the biggest challenge. Firstly, we don’t have the required transport. Sometimes a person’s address has been falsely stated. So even though they are there in that area we cannot find them. Some are reluctant to share this information with us. I’m not sure why but even when we call and ask them they do not say what their true address is.

At times, the time taken to complete the procedures preceding the PHIs visits would adversely affect the surveillance process as the risk factors would have become obsolete or the patients have recovered.

In the words of PHI 12:

…50% there is a delay, mostly in the sending of reports. When we get the report the breeding places may not exist anymore (and) maybe the patient might have been relocated after getting better even (*sic*).

An integral procedure followed by the PHIs during their client visits is to engage them and obtain details for completing 2 forms: the CDF and the DIF, which are both World Health Organization–approved protocols. The sheer length of these forms, which commanded anywhere between 20 and 45 minutes of the PHI’s visits time, apart from its paper-based format requiring manual entry made these procedures prone to error and logistical inconvenience.

Commenting on the imminent risks of losing critical health data from these forms, PHI 13 noted:

We need a lot of space to store these…and if some physical damage happens like mice getting to the forms, the information can be lost.

Finally, PHIs were educating dengue-affected individuals and families using outmoded means of health communication such as pamphlets and brochures. With minimum persuasive impact and lack of audience engagement, PHI reported that these materials bore minimum effects of attitudes, knowledge, and behaviors related to dengue.

#### Technological Challenges

An integral part of the PHIs’ duties during client visits is identification of breeding sites and recording information about them that can be used for follow-up actions such as fogging. However, the difficulty of the dense and unstructured urban terrain in and around Colombo meant that the geographical information recorded by the PHIs suffered on accounts of accessibility and accuracy.

In 2011, the CMC initiated a technological intervention to address this problem by collaborating with the WHO to procure Geographical Positioning System (GPS) devices. The PHIs would thus be required to carry these devices with them in addition to the rest of the paperwork, and after detecting the particular locality’s geographical coordinates, note it down on their paper forms. Although these devices provided partial reprieve to the PHIs, this intervention started triggering its own set of unique technical challenges. For instance, inconsistencies in recording the geographical locations were bound to occur, as each MOH (overseeing multiple PHIs) was equipped with only one device, resulting in a situation where not all PHIs could be uniformly equipped with the system. Furthermore, the density of the terrain comprising uneven housing patterns, unplanned road layouts, and slum colonies meant that the GPS device was, in many instances, unable to procure the signal required to display the precise coordinate of breeding sites. PHI 9 explained the situation thus:

Sometimes I can’t get GPS points. … Sometimes, we have to go like 50 meters away in order to get a significant difference in the GPS coordinates...

In addition, the back-end data management system for the GPS device was set up in a way that required the coordinates captured to transmit through multiple points before finally getting recorded, thereby causing inordinate delays in the data collection process. Pointing out the disadvantage of such delays to breeding site surveillance efforts, PHI 14 noted:

When we give GPS points they form clusters on the central map. Then we can pinpoint breeding places on the map. When we do this in the current system the data has to go here and there and the delay may take days and the breeding place will have served its purpose already.

Commenting on the inability of the existing GPS systems to equip the PHIs with visual maps that can inform them about where dengue outbreaks are occurring, PHI 21 said:

…we don’t know how mapping must be done exactly. If it’s with us then we can know which areas are more prone to cases. But it’s just that we refer record books. We don’t get the mapping information. Although we get the waypoints, we don’t get the resulting mapped data. That does not come to us. We have no feedback from this.

Public health regulations in Sri Lanka allow for legal action to be taken against offenders who fail to address the problem of breeding sites in and around their homes or construction sites (in case of faulty builders). Although the current arrangements allowed the PHIs to record and report the geographical coordinates of errant offenders, the PHIs were unable to provide photographic evidence that would bolster the implementation of such punitive actions. Although some PHIs on their initiatives used their phone camera to capture pictures of breeding sites, these images were seldom allowed as official evidence in the court. Compounding the problem was the fact that not all PHIs were equipped with camera phones, thereby potentiating a situation of inconsistent evidence from the health authorities to the courts.

#### Social Challenges

Despite serving as the last and most critical mile of the public health care system in Sri Lanka, PHIs invoke a range of reactions from the communities they serve during client visits. Ranging from fear and caution to resistance and apathy, these reactions can sometimes stymie the efficiency and speed with which they implement their tasks, as it influences the quality of interaction between the PHI and the client in different ways. At the entry stage, when the PHIs have arrived at the clients’ residence and are introducing themselves, they are used to being met with a range of responses. Some clients immediately request the PHIs to take a seat and are keen to share all the information that the latter need so that their disease can be cured, whereas others could make the PHIs feel unwelcome. As PHI 14 says, over time PHIs have developed their own strategies to smoothen their interaction with the client.

After we go to a patient we can’t suddenly start to fill the form. We must become friendly with them. Then only can we get the correct answers from them.

The PHIs’ affable approach can yield limited results especially when clients are requested for their income as part of the demographic assessment in the forms. The PHIs concur that while this question is casually dealt with by residents in lower income neighborhoods; clients in higher income neighborhoods are more cautious and are thus reluctant to share such information. According to PHIs, client attitudes come into the fore especially when it comes to communicating to them about the actual risks of dengue.

PHI 24 shared his experience thus:

Sometimes, when I tell the truth it’s like a joke to them. When we come they say “here comes the dengue (*sic*).” They take the leaflet and just throw it somewhere. They don’t want to read or they don’t like it. They think it’s a joke, what we’re doing.

A number of the PHIs reported that although they frequently complemented the information in the leaflets with verbal explanations about modes of transmission about dengue and how clients could protect themselves. They were confounded by denial and apathy, especially by members from a particular ethnic community who would, at times, not even allow the PHIs into their houses.

In the words of PHI 9:

…I told them ‘You know more than us about how to stop the spread of dengue through the media and all. Yet you cannot feel the shock you feel now when one of your children is at the risk of death.’ I asked them ‘Do you want to hear that your child is dying?’ Then they got frightened.

As part of their community education efforts, the CMC had also created a movie with an attempt to use the power of an audiovisual medium to enhance the appeal of the messaging. However, the PHIs reported that it was challenging to gather community members at one site at which the movie could be played, and that the contents of the movie too were outdated. In essence, although PHIs acknowledged the potential of moving images to better communicate risk messages about dengue, they equally recognized the inherent constraints in effectively bringing this strategy to the public. PHI 21 elaborated on these challenges:

When we show the movie it changes from area to area. If it’s Cinnamon Gardens who can I show the movie to? …. They aren’t interesting and have no storyline. Also, the people in shanties have no time to spend watching this. They are always trying to spend their time to find some money. So watching a film on dengue is the least of their priorities.

#### Identify Opportunities for Technological Intervention Based on Existing Beliefs and Attitudes and Technological Habits and Exposure

As seen previously, our brief survey preceding the interviews had revealed that despite the limited exposure to and experience with mobile phones, the PHIs demonstrated positive attitudes toward these technologies if integrated into their work. The in-depth interviews helped to generate a more nuanced understanding of the rationale behind their attitudes and the multiple ways in which these technologies could ease their work life, while bolstering dengue prevention efforts in Colombo.

On the basis of their knowledge and our description of mobile phone and tablet capabilities, the PHIs concurred that these technologies would assist in facilitating the data transmission, reporting, and collecting processes. Specifically, the PHIs believed that the burden of executing their dengue-related tasks would be substantively alleviated as these technologies could help PHIs to obtain accurate addresses, dispatch reports from the field rapidly, and possibly even obtain maps of dengue cases. These would not only add to the overall efficiency of their daily performance but also fortify their understanding of dengue spread on a real-time basis, useful knowledge that can be transferred to other dengue-related tasks such as health education. Explaining how the Internet-enabled mobile technologies could aid them in responding to and strategizing programs for dengue outbreaks, PHI 12 said:

If we can submit our report to the EPID (epidemiology) unit directly from the site, including the breeding site information and location I think it would be very useful to us since the report would have come through immediately. Then we can quickly identify areas which could prove to be severe in the following months.

In addition to quicker transmission of field reports, other capabilities of mobile phone technologies, such as reminders, could enable PHIs to be reminded of a new patient report that they would be required to follow-up on, thereby allowing them to plan their workdays in advance and in a more time-efficient manner. Many PHIs suggested that one of the main advantages of such technologies would be in delivering dengue education sessions to clients and other community members. PHIs felt that the use of such technologies would be regarded as a social novelty, which, in turn, would arouse curiosity among community members eventually leading to greater engagement. Apart from obtaining a more detailed awareness of dengue, PHIs believed that these technologies bore greater appeal from a social persuasion perspective and could thus help to transform positive attitudes to behavioral performance.

PHI 12 said:

Yes, it’s more effective definitely. People like it better if we can show them instead of just reciting orally. Even in a school if we demonstrate to the children via a drama for example it becomes more effective.

In summary, our assessment revealed that the current systemic practices surrounding dengue surveillance and prevention were beset by a number of challenges, some of which could be partially addressed with the aid of mobile technologies. Foremost, we found that the workflow surrounding PHIs’ client visits was weighed down by unreliable procurement of clients’ addresses, lengthy paper-based form filling prone to environmental risks, and variable accuracy of geographical coordinates recorded by existing GPS devices. After collecting data, the process of transmitting and processing it through various reports was time consuming and adversely affecting the timeliness of surveillance operations. **Finally**, PHIs’ delivery of dengue education was stymied by outdated modes of communication such as pamphlets and dated movies. However, the PHIs seemed open to and enthusiastic about an **I**nternet-enabled mobile technological intervention albeit some cautious signals from the older PHIs. Overall, the sentiment we gleaned from the interviews seemed to suggest a number of advantages to such an initiative and the PHIs offered concrete ideas that we could built into our innovation, the details of which are presented in the following section.

### Mo-Buzz: A Socially-Mediated System for Dengue Surveillance, Engagement, and Education

The needs assessment helped to identify the key gaps and constraints in the existing dengue information flow and also opportunities to address these using mobile social media. The challenge was to facilitate easier and more efficient exchange of information between actors without changing the existing workflow that has been established according to national guidelines. Instead of digitally transforming the information flow in its entirety, the priority for our innovation would be to address the bottlenecks in steps 8 and 9 identified in Figure 1 and to facilitate a more effective and efficient client visit by the PHIs. The following sections describe our proposed solution, namely Mo-Buzz, which is a socially mediated system that is built on integrated information architecture and is available for PHIs on portable tablets.

### Development Approach

Our approach to developing a social media–based solution for addressing the dengue prevention gaps in Colombo was inspired by the Spiral Model of software development [19-21]. This model is premised on a cyclical notion of software development where “risks” attributed to the system might be incrementally reduced through an iterative, evolutionary process of technical refinement that involves concurrent collaboration between multiple stakeholders. Risks are defined as “situations or possible events that can cause a project to fail or meet its goals” [21].

**Figure 2:**
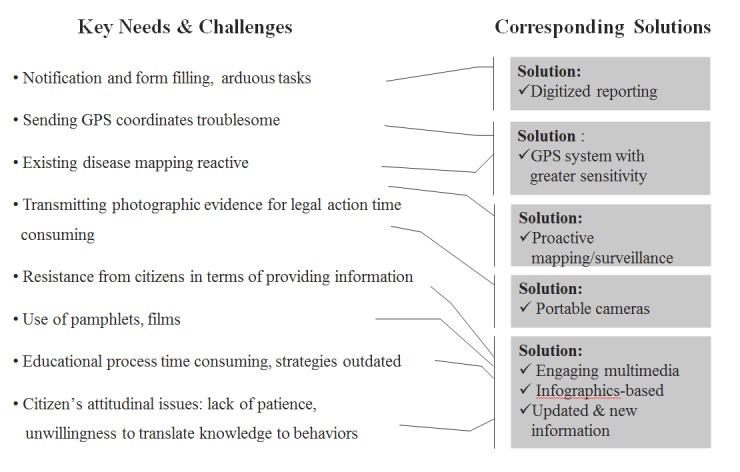
Translation of research findings from needs assessment into mobile solutions.

The conceptual alignment of the Spiral Model with our goals for this study are clear as will be demonstrated by an explanation of the model’s founding ideas. First, the model recommends that the software development process commence by *determining objectives and identifying constraints*, as has been accomplished by our needs assessment. Second, *risks* in the context of a technology-based solution dengue surveillance and prevention in Colombo—a context where PHIs have minimal exposure to smartphones—might pertain to slow or gradual adoption of the technology by PHIs, the delay involved in managing both, the paper-based forms *and* tablets for a period, and the accuracy of the new technology’s reading of the geographical coordinates of a location. Third, involvement of *multiple stakeholders* in the process of developing this technological solution was imminent as any system developed by the research team based in Singapore would need to incorporate the local technical nuances of Colombo, insights that we could best gain by collaborating with institutions such as the CMC, Mobitel (the second largest telecom operator in Sri Lanka), and the University of Colombo School of Computing. Finally, the *evolutionary nature* of technological development was expected because of 3 factors: (1) the system would undergo multiple iterations as PHIs’ comfort and familiarity with the technology increased with use over time, (2) the PHIs would receive feedback from their clients on an ongoing basis, which would need to be incorporated into system refinements, and (3) given that the new system would be developed in parallel to the existing system, we would first need to replicate the existing workflow and then attempt to abbreviate and enhance the process in subsequent versions. Figure 2 graphically depicts how the core needs identified through our research were mapped to potential mobile media solutions.

### Technical Specifications

Our system is built on open source technologies and is mainly purposed for mobile and Web-based application which can be accessed through an Android platform (which eventually be extended to include iOS) or a Web browser. The Android solution forms part of the main application by running as an agent on mobile devices. The PHIs and MOH can report information in various forms (photo or text) using mobile devices. The Web-based solution is designed mainly for the management as it offers an interactive system for geospatial visualization, reports for reported DIF, summaries and graphs, and Web forms for other details. The solution is developed using Java-related technologies. The server side of this system is supported by Apache, Tomcat, and MySQL.

### System Description

The Mo-Buzz system digitizes 3 main functions of PHIs and presents the capability on handheld mobile devices and Web interfaces: (1) capturing, storing and recording visual, textual, and geographical information from patient visits and house or area inspections, (2) staying updated of dengue spread patterns in the Colombo region on a real-time basis, and (3) providing dengue education to the public in an engaging format that will retain their attention and interest.

#### Digital Surveillance

**Figure 3:**
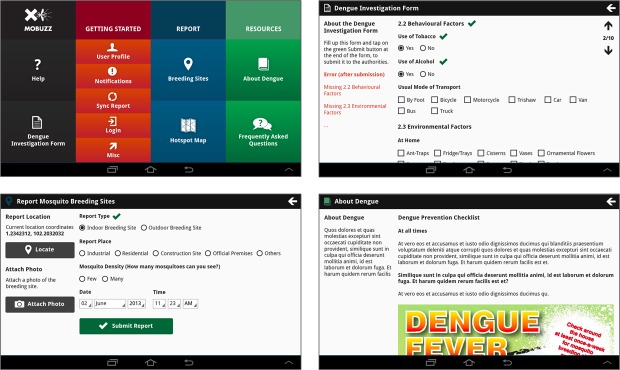
Screenshots from Mo-Buzz depicting the home screen (top left), mosquito reporting form (top right), potential breeding site submission form (bottom left), and health educational component (bottom right).

As seen in Figure 3, this component allows the PHI to capture clients’ information on a digitized DIF form, which is easy to use and includes alerts in case the PHI has missed filling out certain fields. The system thus ensures that the DIF forms are not only complete but also are stored for later reference and can be sent to all the relevant authorities in the different agencies (see Figure 1) with the click of a button, thereby drastically reducing reporting time. In addition, the DIFs are automatically linked to Google Map, thus bolstering every individual DIF with accurate geographical coordinates that can be reviewed by the authorities. The main advantage of this functionality is that the authorities can view, on a continuous, real-time basis, the geographical areas from where dengue cases are being reported and take swift action instead of waiting for paper-based reports to arrive in a delayed manner. This component also allows the PHI to capture photographs of breeding sites, which are automatically geotagged, and share it with all relevant authorities in the chain of command to view and take necessary action (such as fogging and pest control).

#### Digitized Dengue Monitoring and Mapping

In contrast to the CMC’s existing manual pin-maps that can only be updated at the end of every case reporting cycle, the Mo-Buzz system offers a live real-time dengue map that is updated as and when PHIs submit a DIF form to the system. This allows the CMC’s public managers to obtain real-time updates of dengue spread and allocate dengue prevention and management resources strategically and efficiently. This component also automatically draws information from the geotagged breeding site reports and represents this information visually in a map format so that the MOH and their respective PHIs can plan their prevention activities accordingly.

#### Digitized Dengue Education

To increase engagement between the PHI and their clients, the Mo-Buzz system offers a tablet-based health education component. This has been done in the backdrop of mounting evidence that suggests positive outcomes resulting from mobile-mediated health education modules for health workers in other contexts. The first version of the health educational module includes digitized versions of the CMC’s dengue education materials that the PHI presents to his clients complemented by verbal explanations of dengue prevention concepts. We have enhanced the contents with more graphical elements presented in 3 languages (English, Sinhalese, and Tamil) to create awareness among communities with varying levels of linguistic proficiency. For future versions, we are taking the information we gathered to build enhanced graphics, animations, and tailoring capabilities into the health education component.

### Addressing Risks Through an Iterative Process

Mo-Buzz was developed through a series of iterative steps carried out in continuous collaboration with the CMC management, PHIs, and Mobitel. Consistent with the spirit of the Spiral Model, we chronicle the 3 main “risks” that we encountered and explain how these risks were gradually alleviated in subsequent iterations of the system.

The major challenge for the Mo-Buzz system design was the adoption by its target user-base. Most staff members in CMC have not had an experience with digital devices. At the same time, the daily information collection need was substantial.

Our final system was developed through several versions and tested with stakeholders. Each version's initial scope was selected based on feedback from younger PHIs. The rationale was engaging younger staff members as early adopters and eventually ambassadors before extending to older staff.

User interfaces were designed based on CMC's standard documents and followed similar format for simpler forms to help PHI's to get familiar. They were designed to minimize the navigation depth, reduce tedious typing, and all contexts were grouped according to usage patterns of PHIs. The system was introduced with task-oriented trainings, selecting small groups of staff members based on their roles.

#### Risks of Technological Adoption and Change Management

Despite the enthusiasm for mobile technology–based solutions expressed by PHIs and their self-confidence in the ability to handle such solutions, we anticipated challenges in terms of adoption given that our intervention would be completely new to the PHIs’ context. As expected, we gradually discovered that while some PHIs displayed lesser technological skills than we expected, the senior PHIs (by age) were resistant to adopt this new system after years of using paper-based methods. We addressed this risk through a 3-pronged, ongoing strategy. First, the research team constantly consulted with PHIs in the process of development, thereby enhancing their familiarity with the system’s capabilities, and softening their resistance to adopt it. Second, the research team, in collaboration with the CMC management, conducted a number of training sessions that ingrained in PHIs the advantages of the system, and the mechanisms by which our solution could address their daily dengue surveillance concerns. Third, the previously mentioned 2 strategies allowed us to identify specific resistance points within the technology—for instance, the number of fields in the DIF—that we collaboratively managed to reduce over time, maintaining only the most critical informational fields.

#### Structural Risks of Technological Implementation

The technical configuration of our initial system—such as information transfer rate and connectivity strength—was based on our understanding of the strength of mobile technological infrastructure in Colombo, as gleaned from our conversations with experts from CMC and Mobitel, and industry reports. When this version was tested in the controlled environment of Mobitel’s offices, we found minimal inconsistencies with our technical expectations. However, when we used the technology in the field (where PHIs would eventually use it), we discovered a number of issues that needed to be ironed out including weak transfer rates and intermittent mobile connectivity. To overcome these problems in a context where PHIs could least afford to lose data from the field, we developed a simple mechanism that would enable them to save completed DIF forms in case they were unable to immediately send it to the CMC due to network connectivity issues. The system would give them options to retry sending or save temporarily and automatically synchronize at a later time when network connectivity was more stable. Similarly, we discovered that it became challenging to send reports of breeding sites with pictures (in an unstable network connectivity environment) as the images comprised large file sizes. The subsequent iterations of Mo-Buzz involved a mechanism that would automatically resize the images to fit the device’s screen dimensions without severely compromising on picture quality while reducing file size.

#### Health Education Materials on Mo-Buzz

In its incipient stages of development, the Mo-Buzz system comprised digitized versions of existing paper-based health education materials, as desired by the CMC and PHIs. Over time, it became clear that this strategy needed to be revised on account of the simple reason that the multimedia capabilities of tablets were left unused. In subsequent iterations, we collaborated with a team of designers to develop infographics-based health education materials, now available in English, Sinhala, and Tamil and are in the process of incorporating an animation-based dengue education video that the PHIs can show to their community members. We are also in the process of further developing the educational module in a manner that will offer PHIs informational cues and/or alerts in case they fail to cover any important subtopics related to dengue during their interaction with client communities.

We chronicled a range of other risks including issues of linguistic proficiency, range of supported devices, incorporating informational requirements of the CMC management, but have restricted our discussion to 3 risks in the interest of parsimony.

## Discussion

### Principal Findings

Recognizing the severity of the dengue situation in Sri Lanka, our study aimed to identify specific gaps and challenges in surveillance and prevention efforts and use this understanding as the foundation to build an mHealth intervention for PHIs in Colombo. Although our effort was inspired by emerging global efforts using the power of mobile technologies to address public health concerns in developing countries, our objective was to integrate the affordances of multiple solutions and offer them on a common platform, in the form of Mo-Buzz, thereby generating a holistic solution.

Previous studies [22,23] investigating the needs of dengue-related vector-control programs focused on larger systemic issues ranging from insufficient budgets and personnel to challenges in community engagement and interagency collaboration. These findings influenced our intervention to the extent that it fortified our understanding about the potential constraints and organizational challenges that we were likely to confront. The strength of our intervention, however, lies in the ability to acknowledge that while it would be unrealistic to expect mobile technologies to engender a complete systemic transformation, its greater value would lie in being introduced at specific points in the information flow where the tension was greatest. As a result, Mo-Buzz has been designed specifically to address the bottlenecks in steps 8 and 9, where the human elements of communication and action create unique vulnerabilities from a surveillance standpoint. As such, our needs assessment revealed that if the entire dengue information flow were to be metaphorically considered as a sand clock, steps 8 and 9 could be characterized as its neck—the very part that dictates the speed and flow of the process. In other words, the entire informational process curves into and out of the PHI–client interaction.

Recently Labrique et al [24] responded to a persistent criticism about the excessive tendency to report findings from mHealth pilot studies [25], sometimes referred to as “pilotitis,” by highlighting emergent, more scientifically robust, mHealth evidence. Our study is uniquely positioned to contribute to this discussion as we generate empirical evidence about the informational and technological needs of health workers in the context of an mHealth intervention. With respect to the specific concern about piloting, our project, through a collaborative, iterative process of software development, was able to effectively complement the traditional pilot approach, and ensure that all 55 PHIs in the CMC (the entire PHI workforce) gradually adopted the system concurrently. It is important to note here that the size of the PHI workforce, the urban nature of Colombo’s terrain, and the cooperative nature of CMC’s management were factors that facilitated the scale adoption of Mo-Buzz; however, this approach might be less feasible in other public health contexts such as that of the Accredited Social Health Activists in rural India where mHealth innovations need to be introduced in concert with a hierarchical, bureaucratic public health system that is spread out over a vast geographical region. Another focus of Mo-Buzz was to initiate it into the work lives of PHIs in a manner that would cause least disruption to the flow of their daily activities. Although we partially achieved this goal, concerns surrounding the simultaneous management of both, the old paper-based system and the new tablet-based system (Mo-Buzz) continue to exist. These issues will likely be completely ironed out once the paper-based system is fully replaced by a digital version. Similarly, although we continue to work with CMC to formalize the digital reports sent by the PHIs through the tablets, the existing conventions demand added manual tasks, such as a hand-written signature on all completed forms. As we strategize a gradual transfer of technology to CMC, it remains to be seen whether organizational will can be matched with support in terms of funding and technical expertise to streamline organizational processes. Finally, latest reports show large spikes in PHI adoption of Mo-Buzz, to overcome traditional fidelity to paper-based logbooks and handwritten signatures. We expect that over time, more PHIs will be habituated to Mo-Buzz. Contrastingly, we were encouraged to note from some of the PHIs that they were beginning to use the tablets for personal use and other work tasks apart from dengue, such as capturing and storing pictures, sending emails, and so forth. We anticipate that these habits will bear spillover effects for specific use of Mo-Buzz for dengue-related tasks and increase its effective adoption in future.

### Conclusions and Future Work

The Mo-Buzz intervention was first soft launched in June 2013 among a small group of PHIs chosen by the CMC management following which the project team worked on multiple iterations over the next year and half. The system has been fully adopted by the CMC in early 2015 for use by all PHIs. Even so, we expect a gradual adoption curve given the entrenched nature of existing systems in PHI’s work habits. Equally, we also expect variable adoption of the system with respect to its specific components and specific PHI subgroups (younger vs older).

The Mo-Buzz intervention is a response to multiple calls by researchers and the policymaking community for collaborations in the area of mobile interventions for global public health. Our experience revealed that the benefits of this paradigm lies in alleviating country-specific public health challenges through a commonly shared understanding of cultural and ethnic mores and sharing of knowledge and technologies. In the next phase, the research team plans to conduct a theoretically informed, mixed-methods evaluation to assess adoption effectiveness and system performance and its effects on dengue program management metrics of the CMC. We also plan to quantitatively compare the user experiences of the Mo-Buzz system with its paper-based predecessor. We call upon future researchers to further dissect the applicability of the Spiral Model of software development to mHealth interventions and contribute to the mHealth evidence debate from theoretical and applied perspectives.

## Abbreviations

CDF: Communicable Disease Form
CMC: Colombo Municipal Council
DIF: Dengue Investigation Form
EU: Epidemiological Unit
GIS: Geographical Information Systems
GPS: Geographical Positioning System
PHI: Public Health Inspector
